# Association of Serum Prolactin With Type 2 Diabetes Mellitus: A Comparative Cross-Sectional Study From South India

**DOI:** 10.7759/cureus.23721

**Published:** 2022-04-01

**Authors:** C.A. Jayashankar, Akshatha Manohar, Amey Joshi, Vignesh Dwarakanathan, Venkata Bharat Kumar Pinnelli, Vijaya Sarathi, Lakshmi Meghana Gada

**Affiliations:** 1 Internal Medicine, Vydehi Institute of Medical Sciences and Research Centre, Bangalore, IND; 2 Community Medicine, All India Institute of Medical Sciences, New Delhi, IND; 3 Biochemistry, Vydehi Institute of Medical Sciences and Research Centre, Bangalore, IND; 4 Endocrinology and Diabetes, Vydehi Institute of Medical Sciences and Research Centre, Bangalore, IND

**Keywords:** south indian, glycaemic indices, serum lipid profile, serum prolactin, type 2 diabetes mellitus

## Abstract

Background: The association of serum prolactin (PRL) with diabetes is still uncertain, with a paucity of data in the south Indian population. This study aims to compare the serum PRL levels between type 2 diabetes mellitus (T2DM) patients and normoglycaemic volunteers and correlate the serum PRL level with fasting plasma glucose (FPG), postprandial plasma glucose (PPG), glycated haemoglobin (HbA1c) levels, and the lipid profile in the study population.

Methods: This was a comparative cross-sectional study among 112 T2DM participants and 112 healthy volunteers in a tertiary care centre in India. All participants were tested for FPG, PPG, HbA1c, fasting serum lipid profile, and serum PRL, which were compared between T2DM patients and healthy volunteers.

Results: The serum PRL in T2DM patients was significantly lower compared to healthy volunteers (8.67 ± 4.37 vs. 13.76 ± 6.55 ng/ml, P < 0.001). FPG, PPG, and HbA1c correlated inversely with serum PRL in our study population. On multivariable logistic regression adjusted for age and sex, a higher serum PRL level within the physiological range was protective for T2DM (adjusted odds ratio: 0.83, 95% CI: 0.77-0.90, P < 0.001). Serum PRL levels were inversely correlated with serum total cholesterol, low-density lipoprotein cholesterol, and triglycerides, but not with high-density lipoprotein cholesterol.

Conclusions: A high serum PRL within the physiological range was inversely associated with the prevalence of T2DM in the south Indian population. Serum PRL also correlated inversely with glycaemic and blood lipid parameters. Larger longitudinal studies are required to further validate the association of serum PRL with various components of metabolic syndrome in the south Indian population.

## Introduction

The increasing evidence of the diverse functions of prolactin (PRL) has warranted its investigation beyond its recognized role in lactation to study its effect on islet cell differentiation, immune modulation, and adipocyte control. The identification of PRL receptors on various target tissues has accelerated research and piqued interest in the pathophysiological effect of this hormone on different organ systems [1]. High serum PRL level in the physiological range was found to protect against type 2 diabetes mellitus (T2DM). On the contrary, high serum levels of this hormone in pathological settings increase the risk of developing T2DM [2]. Cellular studies now reveal that PRL activates pancreatic β-cells and reduces the glucose threshold for insulin secretion through various signalling pathways [3,4]. A recent meta-analysis demonstrated that higher PRL levels within the normal range reduced the risk of prevalent T2DM but not incidental T2DM, thus leaving us with limited evidence of its causal association [2].

This study was undertaken to assess PRL levels in patients with T2DM and healthy volunteers. The high incidence of T2DM in the south Indian population also necessitates further investigation of the association of PRL and glycaemic parameters in this geographical landscape. As a secondary objective, we also compared the different lipid parameters and their association with varying PRL levels.

## Materials and methods

The study conformed to the principles of the Declaration of Helsinki and was approved by the Vydehi Institutional Ethics Committee (VIEC/2017/APP/107). Written informed consent was obtained from all the participants in the local language.

This was a single-centre, comparative, cross-sectional study conducted at a tertiary care hospital in Bangalore, India between January 2018 and July 2019. Participants were selected from the outpatient clinic and inpatient services under the department of general medicine. For estimating the sample size, we considered a mean difference in serum PRL of 1.5 ng/mL between patients with T2DM and healthy volunteers, standard deviations of 3.6 and 2.4 ng/ml [5], alpha error of 5%, and power of 90%. We assumed a non-response rate of 20% and estimated our sample size to include 112 patients with T2DM and 112 healthy volunteers.

The study group consisted of adult (age > 18 years) T2DM patients diagnosed according to the American Diabetes Association criteria [6]. The control group consisted of healthy volunteers (age > 18 years) who were not diabetic or pre-diabetic according to the aforementioned diagnostic criteria. Pregnant or lactating women, patients with type 1 diabetes mellitus, or those with elevated serum PRL levels greater than or equal to two times the upper limit of normal were excluded from the study. Furthermore, those patients on active or past treatment of prokinetics, corticosteroids, thyroid hormones, antipsychotics, selective serotonin reuptake inhibitors, oral contraceptive pills, or statin therapy were excluded from the study to minimize confounding bias arising due to the potential effect of these medications on serum PRL levels.

All participants were tested for glycated haemoglobin (HbA1c), fasting plasma glucose (FPG), postprandial plasma glucose (PPG) two hours after a regular meal, and serum PRL. The participants were also tested for fasting lipid profiles, which included total cholesterol (TC), low-density lipoprotein cholesterol (LDL-C), high-density lipoprotein cholesterol (HDL-C), and serum triglycerides (TG). HbA1c was measured by the high-performance liquid chromatography method and plasma glucose was measured by the glucokinase method. Fasting serum PRL was measured by the chemiluminescence method. Serum TC was measured by the cholesterol oxidase esterase method, TG by enzymatic glycerol phosphate oxidase method, and HDL-C and LDL-C by direct measure polymer method.

Data were analysed using Stata version 12 (StataCorp LLC, College Station, TX) and were presented as frequencies and proportions for categorical variables, and as mean and SD for continuous variables. Wilcoxon rank-sum test was applied to test the difference between two continuous variables. The chi-square test or Fischer's exact test was used as appropriate to test distributions for categorical variables. Quartiles of serum PRL were generated separately for males and females. Glycaemic parameters (FPG, PPG, and HbA1c) were measured across these quartiles of serum PRL separately for males and females, and the trend was tested by the Cuzick test (1985). Pearson’s correlation coefficient was calculated between serum PRL, glycaemic parameters, and lipid profile for the total population. Multivariable logistic regression was done to find the association of serum PRL with T2DM after adjusting for age and sex.

## Results

A total of 112 patients diagnosed with T2DM and 112 healthy volunteers were enrolled in our study. The patients with T2DM were significantly older than the control group. Serum TC, LDL-C, HDL-C, and TG were found to be significantly higher in the patients with T2DM (Table 1). We observed that the patients with T2DM had a significantly lower level of serum PRL than healthy volunteers (8.75 ± 4.37 vs. 13.76 ± 6.5, P < 0.001).

**Table 1:** Demographic, clinical, and laboratory characteristics of participants. Continuous variables are presented as mean ± standard deviation. Categorical variables are presented as frequencies and percentages.

Variables	Type 2 diabetes mellitus (n = 112)	Healthy volunteers (n = 112)	P-value
Demographic and clinical characteristics			
Age (years)	48.88 ± 9.09	37.91 ± 12.53	<0.001
Female (n, %)	46 (41.1%)	51 (45.5%)	0.514
Systolic blood pressure (mmHg)	131.16 ± 17	120.11 ± 17.14	<0.001
Diastolic blood pressure (mmHg)	82.53 ± 9.3	74.41 ± 9.6	<0.001
Laboratory parameters			
Fasting plasma glucose (mg/dl)	214.98 ± 95.56	84.19 ± 17.44	<0.001
Postprandial plasma glucose (mg/dl)	318.67 ± 124.91	107.32 ± 23.31	<0.001
Glycated haemoglobin (%)	10.09 ± 3.01	5.38 ± 0.46	<0.001
Serum total cholesterol (mg/dl)	187.95 ± 60.55	151.98 ± 60.14	<0.001
Serum low-density lipoprotein cholesterol (mg/dl)	123.05 ± 48.09	94.22 ± 45.36	<0.001
Serum high-density lipoprotein cholesterol (mg/dl)	39.18 ± 12.38	35.09 ± 13.81	0.01
Serum triglycerides (mg/dl)	190.72 ± 86.25	152.86 ± 72.43	0.001
Serum prolactin (ng/ml)	8.67 ± 4.37	13.76 ± 6.5	<0.001

To evaluate the association of serum PRL and glycaemic parameters (FPG, PPG, and HbA1c) in males and females separately (N = 127 and N = 97, respectively), we divided our entire sample to quartiles with different cut-offs based on serum PRL levels (males - Q1: <7.2 (n = 31), Q2: 7.3-9.5 (n = 32), Q3: 9.6-12.6 (n = 31), and Q4: >12.6 (n = 33); females - Q1: <6.6 (n = 23), Q2: 6.7-10.4 (n = 24), Q3: 10.5-14.9 (n = 25), and Q4: >14.9 (n = 25)). We observed a significant decreasing trend of PPG, FPG, and HbA1c levels irrespective of gender with increasing serum PRL levels (Table 2).

**Table 2:** Comparison of the glycaemic parameters among all the participants in serum prolactin-based quartiles. All values are expressed as median (interquartile range). Prolactin quartiles in males (ng/ml) - Q1: <7.2, Q2: 7.3-9.5, Q3: 9.6-12.6, and Q4: >12.6. Prolactin quartiles in females (ng/ml) - Q1: <6.6, Q2: 6.7-10.4, Q3: 10.5-14.9, and Q4: >14.9.

	Men				
	Quartile 1	Quartile 2	Quartile 3	Quartile 4	P-value for trend
	n = 31	n = 32	n = 31	n = 33	
Prolactin (ng/ml)	<7.2	7.3-9.5	9.6-12.6	>12.6	
Fasting plasma glucose (mg/dl)	215 (104.5-278.5)	106 (87-154.5)	100 (75-139)	102 (78-134)	0.001
Postprandial plasma glucose (mg/dl)	316.5 (173-424.5)	193.5 (116.5-245)	126 (96-249)	134 (101-225)	<0.001
Glycated haemoglobin (%)	9.9 (6.45-12)	7.05 (5.6-8.6)	5.6 (5.2-8.5)	5.7 (5.5-7.1)	<0.001
	Women				
	Quartile 1	Quartile 2	Quartile 3	Quartile 4	P-value for trend
	n = 23	n = 24	n = 25	n = 25	
Prolactin (ng/ml)	≤6.6	6.7-10.4	10.5-14.9	>14.9	
Fasting plasma glucose (mg/dl)	168 (111-252)	177.5 (94-238)	88 (76-102)	82 (75-92)	<0.001
Postprandial plasma glucose (mg/dl)	261.5 (191-379.5)	275.5 (142.5-341.5)	104 (90-150)	106 (89-134)	<0.001
Glycated haemoglobin (%)	8.7 (6.5-10.1)	8.65 (5.7-13.8)	5.7 (5.2-6.9)	5.3 (4.9-5.7)	<0.001

In our entire cohort (n = 224), serum PRL level negatively correlated with FPG (r = −0.28, P < 0.01), PPG (r = −0.34, P < 0.01), HbA1c (r = −0.31, P < 0.01), TC (r = −0.24, P < 0.01), LDL (r = −0.21, P < 0.01), and TGs (r = −0.19, P = 0.01).

After adjusting for age and gender, on multivariate logistic regression, we observed that serum PRL was independently associated with T2DM (adjusted odds ratio: 0.83, 95% CI: 0.77-0.90, P < 0.001). Odds of having T2DM decreased by 17% with each unit increase in serum PRL (ng/mL) within the normal range (Table 3).

**Table 3:** Multivariable logistic regression of factors associated with type 2 diabetes compared to healthy volunteers.

	Crude odds ratio	P-value	Adjusted odds ratio	P-value
Serum prolactin (ng/ml)	0.82 (0.76-0.88)	<0.001	0.83 (0.77-0.90)	<0.001
Age (years)	1.09 (1.06-1.12)	<0.001	1.08 (1.05-1.12)	<0.001
Male sex	1.20 (0.71-2.03)	0.500	1.10 (0.58-2.09)	0.760

## Discussion

In this cross-sectional study involving south Indian patients with T2DM and healthy volunteers, we found a negative association of glycaemic parameters with serum PRL levels irrespective of gender. We also noted that the odds of having T2DM decreased by 17% with every one unit increase of serum PRL when within the physiological range.

The role of PRL in the initiation and maintenance of lactation has been established; yet, newer insights into its role in various organ systems and metabolism continue to emerge. The PRL receptor is expressed on hair follicles, gastrointestinal tracts, retina, adipose tissue, and pancreatic beta cells [1]. This expression of the PRL on pancreatic β-cells was postulated to play a role in β-cell proliferation during pregnancy to meet the increased metabolic demands [7]. This was later confirmed by in vivo studies wherein PRL receptor-deficient mice had reduced β-cell mass and density. This led to 20-35% less insulin production by islets, ultimately making the mice glucose intolerant [3]. PRL-treated cells were found to have an increased expression of glucokinase enzyme, a rate-limiting enzyme in glucose metabolism. As a downstream effect, the PRL-treated islet cells could produce insulin at a faster rate [1]. To test this theory, numerous studies have been undertaken to understand the clinical implication of PRL in T2DM in humans.

A large meta-analysis involving six studies from 2013 to 2019 demonstrated high PRL levels in the physiological range to be associated with a lower prevalent T2DM [2]. A study by Wang et al. divided their study population into quartiles based on serum PRL levels similar to our study. They observed men with a serum PRL level > 10.63 ng/ml and women with a serum PRL level > 11.50 ng/ml had an adjusted OR of 0.38 (0.24-0.59) and 0.47 (0.32-0.70) of having T2DM, respectively, with Q1 referent [8]. A cross-sectional study by Chahar et al. concluded that high serum PRL levels within the physiological range were associated with reduced odds of T2DM in women but not in men (OR: 0.13 (0.03-0.56) vs. OR: 0.61 (0.21-1.72)) [9]. A 22-year follow-up study in the United States studying serum PRL in women revealed that high levels of PRL reduce the risk of incident T2DM ((HR: 0.73 (0.55-0.95)) [10]. This finding was also consistent with another case-control study by Manshaei et al., which reported markedly lower serum PRL levels in females with T2DM than in healthy volunteers (5.32 ± 0.36 vs. 18.38 ± 2.3 ng/dl) [11]. The stronger association in females may be attributed to relatively higher serum PRL levels than males and a protective effect of estrogen on β-cells via anti-inflammatory effects and insulin sensitivity [12]. However, there was no effect-modification by gender in the association of serum PRL with T2DM risk in our study (data not shown). Extending the studies beyond dysglycaemic individuals, a study by Albu et al. reported an inverse relation of serum PRL with FPG among healthy subjects [13]. The observations in the aforementioned studies suggest that the effect of PRL on glycaemic parameters may be less pronounced in dysglycaemic individuals, part of which might be attributed to treatment-related factors such as the PRL-lowering effect of metformin [14].

While these associations are only applicable to higher levels of PRL within the physiological range, high PRL levels in the non-physiological range due to prolactinoma or treatment with antipsychotic drugs have consistently been shown to unfavourably affect metabolic parameters [15,16]. A pathologically higher PRL level in patients with T2DM in a few studies has also been shown to be associated with increased diabetic complications, insulin resistance, and metabolic syndrome [17]. The Framingham Heart Study observed an incremental rise in incident T2DM in the male population with every 5 mg/dl increase in serum PRL levels (OR: 1.70, 95% CI: 1.04-2.78, P = 0.03) [5]. This association, however, was attenuated when the authors adjusted for BMI. In another study involving 3929 patients of which 21% were T2DM patients, a higher all-cause (men - HR: 1.75; 95% CI: 1.32-2.32; women - HR: 1.66; 95% CI: 1.08-2.56) and cardiovascular mortality (men - HR: 2.16; 95% CI: 1.27-3.67; women - HR: 2.84; 95% CI: 1.38-5.89) was observed with higher PRL concentrations; however, this result did not adjust for BMI [18]. These studies were primarily carried out in the Caucasian population and maybe one of the attributing factors for the contradicting results, as observed in the present study.

Notably, recent studies have observed contradicting evidence on the role of PRL in T2DM and thus the temporality of this association has not yet been established. Although in the present study, we observed favourable outcomes of high physiological levels of PRL and lower prevalent T2DM in the sub-set of the south Indian population, longitudinal follow-up studies are required to establish the causality of this association. The current evidence, however, helps us conclude that both low and supraphysiological serum PRL levels unfavourably affect diabetic metabolic parameters.

Our study found that serum PRL had a significant inverse correlation with TC, LDL-C, and TG in the study population, but no significant correlation with HDL-C. Glintborg et al. observed that PRL was positively associated with HDL-C (r = −0.11, P < 0.05) and inversely associated with TC (r = −0.15, P < 0.05) and TG (r = −0.14, P < 0.05), with the inverse association between PRL and LDL-C (r = −0.15, P < 0.05) being independent of age and BMI in a cohort of polycystic ovary syndrome (PCOS) patients [19]. Other studies did not correlate between lipid parameters and PRL [20,21]. PRL receptors are present in adipose tissue, which is involved in its regulation. In vitro studies showed that human adipose tissue treated with PRL caused downregulation of lipoprotein lipase and fatty acid synthase leading to suppression of lipogenesis. It also increased leptin and adiponectin levels and reduced inflammatory markers such as interleukin 1 beta (IL-1β) and interleukin 6 [22]. Conversely, patients with prolactinomas had a lower level of HDL-C and higher levels of LDL-C and TG compared to healthy adults [23,24]. Furthermore, treatment with cabergoline for six months caused significant improvement in the lipid profile with a decline in TC, LDL-C, and TG [23]. The present evidence is insufficient to justify the temporality of serum PRL and serum lipid concentrations due to the direct association of dopaminergic activity on adiposity and lipid levels.

Limitations

The findings of our study have to be interpreted in light of a few limitations. Though we have restricted our population to eliminate a few confounders such as pregnancy, lactation, thyroid disorders, use of statins, PRL secretagogues/secretion inhibitors, and adjusted for sex, residual confounding might be present due to these factors. The higher age of participants in the T2DM group may have led to the inverse correlation of PRL with glycaemic parameters. The effect of the antidiabetic medications being taken by the T2DM individuals on the level of serum PRL could not be evaluated. Although the association between T2DM and PRL was significant, the temporality of this association was not determined in our study owing to its observational design. We could not adjust for insulin resistance or estrogen concentrations in our study.

## Conclusions

T2DM is associated with lower serum PRL levels than those without diabetes mellitus. Serum PRL also had a significant inverse correlation with TC, LDL-C, and TG. These observations suggest a role for PRL in the pathogenesis of metabolic dysregulation among the south Indian population. More extensive multi-centric longitudinal studies are required to validate the temporality of the association between serum PRL and T2DM incidence.
